# Emerging roles for methionine metabolism in immune cell fate and function

**DOI:** 10.3389/fimmu.2025.1701505

**Published:** 2025-12-09

**Authors:** Shaojuan Liu, Xiaotian Tang, Min Huang, Jianian Tang, Yiwen Yang

**Affiliations:** 1 Agricultural Products Processing Research Institute, Chinese Academy of Tropical Agricultural Sciences, Zhanjiang, China; 2 College of Food Science, Fujian Agriculture and Forestry University, Fuzhou, China; 3 South Subtropical Crops Research Institute, Chinese Academy of Tropical Agricultural Sciences, Zhanjiang, Guangdong, China; 4 College of Animal Science and Technology, College of Veterinary Medicine, Zhejiang A&F University, Hangzhou, China; 5 Key Laboratory of Feed Biotechnology of Ministry of Agriculture and Rural Affairs, Institute of Feed Research, Chinese Academy of Agricultural Sciences, Beijing, China

**Keywords:** methionine metabolism, immune cells, autoimmunity, cancer, reprogramming

## Abstract

Metabolic reprogramming is a critical characteristic essential for the activation of immune cells. The altered amino acid metabolism, particularly changes in methionine metabolism, holds significant importance in directing the fate and function of diverse immune cells. Here we summarize the main transport system and metabolic pathway of methionine in immune cells, and the well-established and novel research findings of methionine metabolism-dependent modulation on major immune cell lineages and cancer cells are provided afterward. Unraveling the potential regulatory mechanism of methionine metabolism reprogramming in immune cells provides the new strategies for the therapy of autoimmune diseases and cancers.

## Introduction

The diverse immune cell populations play a critical role in the immune system, being distributed throughout immune organs and performing essential functions in maintaining normal bodily processes and combating pathogen infections. The fate of immune cells is extensively dictated by the metabolic profiles, which can be rapidly altered in response to antigens and cytokines. For instance, macrophages can be categorized into M1/M2 subtypes based on their activation by different stimuli. Inflammatory M1 macrophages primarily rely on aerobic glycolysis and the pentose phosphate pathway to enhance antimicrobial effects, while alternatively polarized M2 macrophages utilize glucose and fatty acid oxidation to coordinate reparative functions (1–3). The metabolic reprogramming is regulated by the availability and accessibility of specific nutrients. As such, the regulatory role of nutrients, as well as their metabolites, on immune cell fate and function has emerged as a prominent area of research in immunology in recent years (4, 5).

Methionine, an essential sulfur-containing amino acid, not only participates in protein biosynthesis but also functions as an intermediate metabolite and signaling molecule involved in redox maintenance and methylation processes (6–8). Immune cells demonstrate specific methionine requirements, with their activation triggering upregulation of methionine transporters to facilitate rapid proliferation and enhance the production of cytokines and adhesion molecules necessary for immune responses (9). Conversely, certain pathological environments such as the tumor microenvironment can significantly impair immune cell functions due to the severe methionine deprivation (10). Remarkably, tumor cells also demonstrate enhanced uptake and metabolism of methionine (11), underscoring the central role of methionine metabolism in tumorigenesis. These characteristics of methionine in immune cells and tumors present a promising therapeutic strategy for autoimmune diseases and cancers.

In this review, we present a comprehensive overview of the gene expression patterns of diverse methionine transporters in major immune cell types, encompassing T lymphocytes, B lymphocytes, macrophages, dendritic cells (DCs), and natural killer (NK) cells. Subsequently, we summarize the intricate networks involved in methionine metabolism within immune cells, including the methionine cycle, transsulfuration pathway, methionine salvage pathway, and catalytic cycle of methionine residues. Furthermore, we discuss the regulatory role and potential mechanisms by which methionine metabolism influences various immune cell types. For example, through its regulation of epigenetic reprogramming, methionine metabolism may shape the T helper (Th) cell response (12). Lastly but importantly, we emphasize the indispensable role played by methionine metabolism during tumorigenesis and evaluate potential strategies for cancer treatment targeting this metabolic pathway. This comprehensive review provides an in-depth understanding of the significance of methionine metabolism in governing immune cell function.

## Methionine transport system in immune cells

Immune cells undergo cellular metabolic reprogramming upon activation by antigens or cytokines, necessitating substantial nutrient uptake to support rapid proliferation and differentiation. Amino acids, as pivotal nutrients for immune cells, exert profound effects on cell function due to their dual role as both fundamental building blocks for protein biosynthesis and crucial signaling molecules (13). To fulfill the escalating demand for amino acid consumption, immune cells rapidly regulate amino acid transporters form solute carrier (SLC) superfamily to facilitate extracellular amino acid uptake (14).

**Figure 1:**
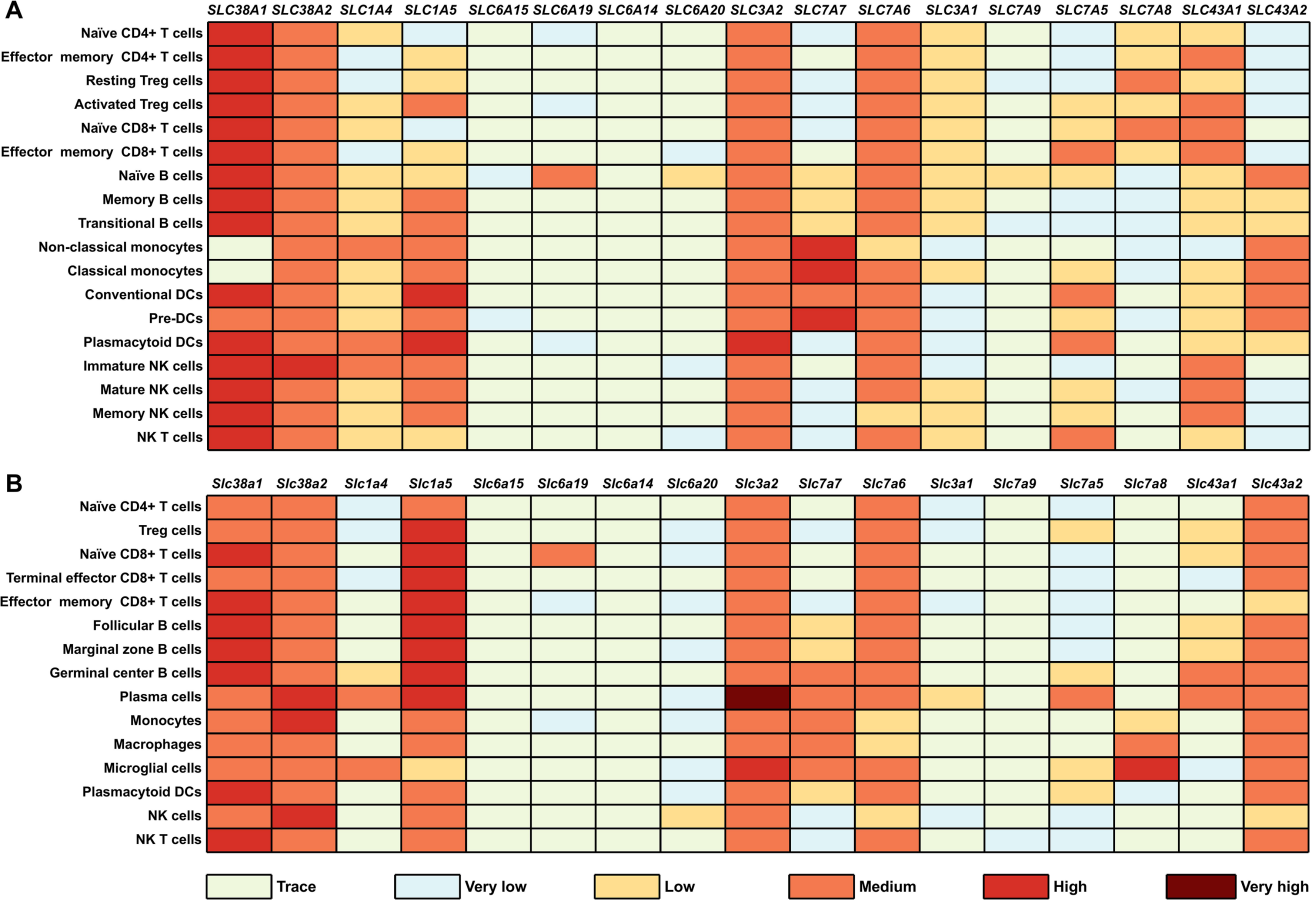
Gene expression patterns of methionine transport in different immune cells. Current knowledge of expression of methionine transporters in different immune cells from **(A)** humans and **(B)** mice. The immune cell populations are sorted based on the specified phenotype, primarily from human blood and mouse spleen. RNA sequencing data are obtained from the Immgen database (https://www.immgen.org) and normalized by DESeq2. The expression value is defined as: trace, 0-5; very low, 5-10; low 20-80; medium, 80-800; high, 800-8000; very high, >8000.

Cellular methionine uptake involves multiple amino acid transporters, including mainly ASCT2 (SLC1A5), B^0^AT1 (SLC6A19), 4F2hc/LAT1 (SLC3A2/SLC7A5), and 4F2hc/y^+^LAT1 (SLC3A2/SLC7A7), with minor involvement of SNAT1 (SLC38A1), SNAT2 (SLC38A2), ASCT1 (SLC1A4), 4F2hc/y^+^LAT2 (SLC3A2/SLC7A6), B^0^AT2 (SLC6A15), ATB^0,+^ (SLC6A14), SIT1 (SLC6A20), rBAT/b^0,+^AT (SLC3A1/SLC7A9), LAT3 (SLC43A1), and LAT4 (SLC43A2) (**Figure 1**) (15–19). Although methionine can be transferred by a broad spectrum of amino acid transporters, the expression levels of these transporters in different immune cell subtypes vary greatly (**Figure 1**). The high-affinity transporter SLC3A2/SLC7A5 is the primary methionine transporter found in most immune cell types, facilitating methionine translocation in a Na^+^ independent manner (18).

Numerous studies have demonstrated the significant role of the SLC3A2/SLC7A5 transporter in methionine uptake by activated immune cells. Stimulation of CD19^+^ B cells with B cell receptor (BCR)/Interferon-α (IFN-α)/CpG leads to a rapid increase in methionine intake through upregulation of SLC3A2/SLC7A5 transporters (20). On the contrary, *Slc7a5*-null CD4^+^ T cells manifest reduced methionine influx and impaired differentiation following T cell receptor (TCR, CD3/CD28) stimulation (9). These findings introduce an essential question regarding the precise mechanism by which activated lymphocytes modulate the expression of SLC3A2/SLC7A5 transporter. In immune cells (T cells, B cells, and nature killer cells), engagement of stimuli (e.g., TCR, BCR/CD40, and IL-2/IL-12) rapidly induces the transcription factor MYC, which subsequently upregulates various amino acid transporters including SLC7A1, SLC7A5, SLC1A5, SLC7A6, and so on (21–24). Knockdown of MYC result in an astonishing reduction of SLC7A5 in both activated CD4^+^ or CD8^+^ T cells (24). Collectively, immune cells sustain MYC expression upon activation, therefore promoting the upregulation of methionine transporters and ultimately increases the methionine uptake.

## Methionine metabolism in immune cells

The rapid proliferation of immune cells is one of the primary hallmarks of immune response to antigen stimulation, and numerous metabolic substrates are required for this process. Hence, methionine metabolism in majority of immune cells is expended for the biosynthesis of protein, the methionine kinetics detection *in vivo* also reveals that it mainly takes place in liver, kidney, brain, and pancreas tissues (25). Methionine (encoded by AUG) is the initial amino acid of the protein biosynthesis in eukaryotic cells, which is loaded onto methionyl-tRNAs by methionyl-tRNA synthetases and guides the synthesis of peptide chain (26, 27). The remaining methionine flux, in addition to its role in protein turnover, enters various metabolic pathways and contributes to the production of multiple functional downstream metabolites.

### Methionine cycle

**Figure 2:**
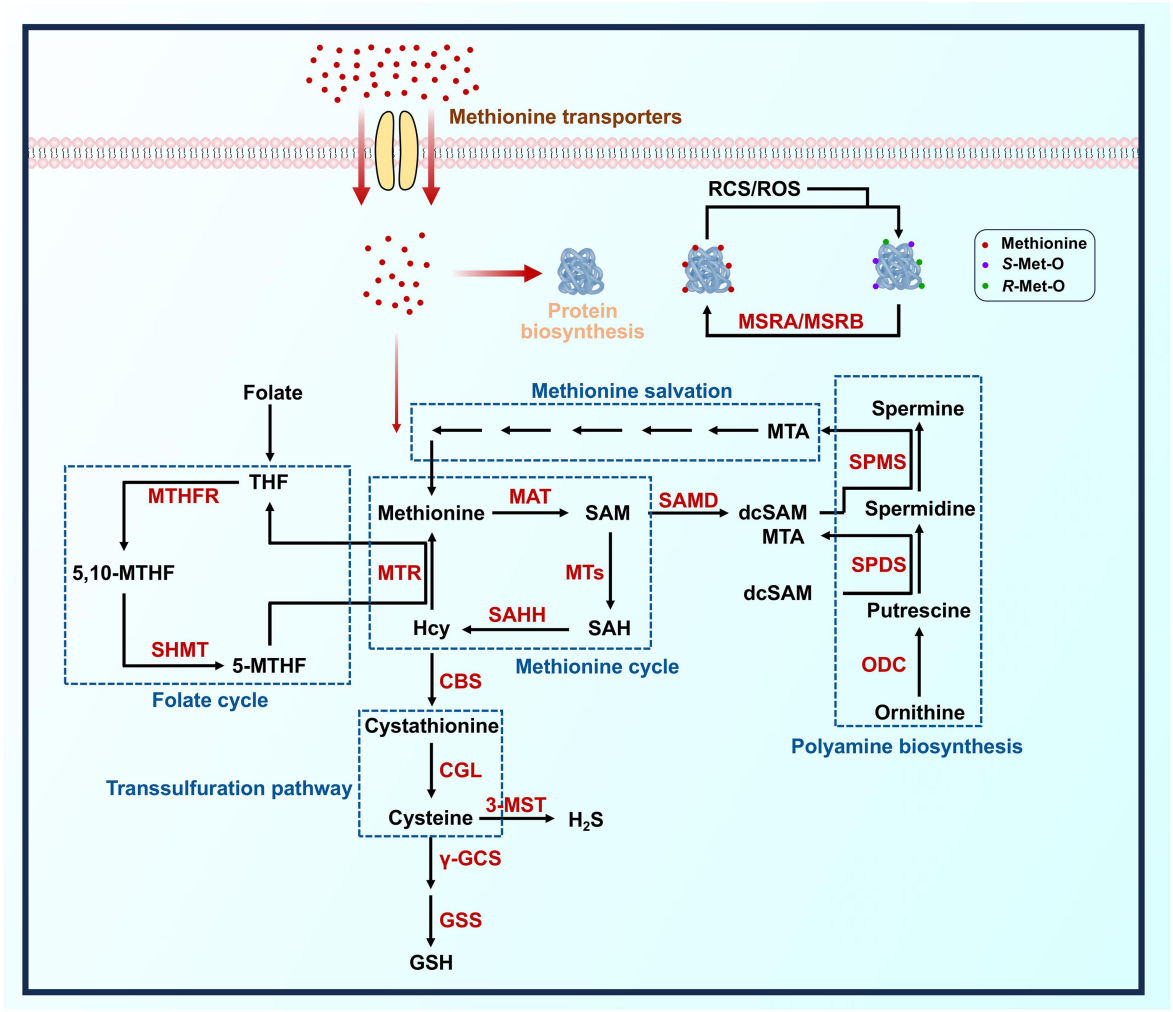
Overview of pathway involved in methionine metabolism for immune cells. Schematic diagram detailing primary pathways for methionine metabolism. The enzymes that catalyze these reactions are highlighted in red. CBS, cystathionine β-synthase; CGL, cystathionine γ-lyase; dcSAM, decarboxylated *S*-adenosylmethionine; GSH, glutathione; GSS, glutathione synthetase; Hcy, homocysteine; H_2_S, hydrogen sulfide; MAT, methionine adenosyltransferase; MSRA, methionine sulfoxide reductase A; MSRB, methionine sulfoxide reductase B; MTA, 5’-methylthioadenosine; MTHFR, methylenetetrahydrofolate reductase; MTR, 5-methyltetrahydrofolate homocysteine methyltransferase; MTs, methyl transferases; ODC, ornithine decarboxylase; RCS, reactive chlorine species; *R*-Met-O, *R*-methionine sulfoxide; ROS, reactive oxygen species; SAH, *S*-adenosylhomocysteine; SAHH, *S*-adenosylhomocysteine hydrolase; SAM, *S*-adenosylmethionine; SAMD, S-adenosylmethionine decarboxylase; SHMT, serine hydroxymethyltransferase; *S*-Met-O, *S*-methionine sulfoxide; SPDS, spermidine synthase; SPMS, spermine synthase; THF, tetrahydrofolate; γ-GCS, γ-glutamate cysteine ligase; 3-MST, 3-mercaptopyruvate sulfurtransferase; 5-MTHF, 5-methyltetrahydrofolate; 5, 10-MTHF, 5,10-methylenetetrahydrofolate.

Methionine cycle represents the central pathway of methionine metabolism, encompassing a series of reactions that both catabolize and regenerate methionine (**Figure 2**). Methionine adenosyltransferases (MATs) serve as rate-limiting enzymes responsible for the conversion of methionine to *S*-adenosylmethionine (SAM). SAM is subsequently converted to *S*-adenosylhomocysteine (SAH) by various methyltransferases (MTs), participating in DNA, RNA, protein, and lipid methylation processes. SAH undergoes hydrolysis mediated by SAH hydrolase (SAHH), resulting in the formation of homocysteine (Hcy). Hcy then receives a methylation group from 5-methyltetrahydrofolate (5-MTHF) through the action of 5-methyltetrahydrofolate homocysteine methyltransferase (MTR), ultimately leading to its remethylation and recycling back into methionine (28, 29).

SAM is considered the foremost biological methyl donor in mammals due to its unique chemical structure, which plays a crucial role in the methylation epigenetics of immune cells (30). The cellular SAM level depends on the activity of MATs. Mutiple MAT isoforms (MAT1A, MAT2A, and MAT2B) have been identified in human immune cells, while only two MAT isoforms (Mat2a and Mat2b) have been identified in murine immune cells (31, 32). SAM-dependent methylation is a known epigenetic mechanism that modulates immune cell function through extensive MTs and produces SAH as the by-product. Notably, MT activity can be inhibited by the feedback from SAH, and thus, the cellular SAM: SAH ratio serves as an indicator of “methylation potential” for evaluating cellular methylation statues (33). Manipulating cellular SAM and SAH levels is sufficient to determine the methylation levels in immune cells. For example, altering the levels of SAM and SAH using 3DAZ, an inhibitor decreases the cellular SAM: SAH ratio, reduces histone methylation of key genes in lipopolysaccharide (LPS)-stimulated macrophages (34). Interestingly, the production of downstream Hcy also influences the cellular SAM: SAH ratio. Inhibitors of SAHH (e.g., tubercidin and DZ2002) have been shown to profoundly affect inflammatory factor secretion and neutrophil migration by influencing the gene methylation modifications (35–37). In the final step of methionine cycle, Hcy acquires methyl groups from either 5MTHF or betaine to produce recycled methionine. However, most types of immune cell highly express MTR rather than betaine-homocysteine methyltransferase (BHMT), indicating that the folate cycle is a critical metabolic pathway for methionine re-production.

### Transsulfuration pathway

In addition to being remethylated back to methionine, approximately half of Hcy pool could be metabolized into cysteine through the transsulfuration pathway (**Figure 2**). Cystathionine β-synthase (CBS) catalyzes the condensation of serine with Hcy in a pyridoxal-5’-phosphate-dependent (PLP-dependent) manner, resulting in the formation of cystathionine. Subsequently, cystathionine is cleaved by cystathionine γ-lyase (CGL) to generate cysteine (38). Further metabolism of cysteine involves enzymatic reactions catalyzed by γ-glutamylcysteine synthetase (γ-GCL)/glutathione synthetase (GSS) and 3-mercaptopyruvate sulfurtransferase (3-MST), leading to the synthesis of hydrogen sulfide (H_2_S) and glutathione (GSH), respectively002E.

Cysteine is the central metabolite in transsulfuration pathway, and the cellular *de novo* synthesis of cysteine is dependent on the transsulfuration activity. Interestingly, the rate of transsulfuration is also determined by the cellular SAM: SAH ratio, while cysteine deprivation induces the upregulation of transsulfuration enzymes (CBS and CGL) through GCN2-ATF4 axis (39). Moreover, cysteine exerts diverse physiological functions by either metabolizing into endogenous gasotransmitter H_2_S or antioxidant GSH (40). Notably, exposure to exhaust particles significantly alters intracellular levels of cysteine and GSH levels in alveolar macrophages and lymph node cells, leading to a severe inflammation in lung (41). Additionally, cysteine also serves as the sulfur donor for synthesizing several iron-sulfur (FeS) clusters, such as RNA Pol-III and Elp3. These clusters play both structural and catalytic roles in supporting the function of [FeS] proteins that recognize viral factors and activate innate immune responses during infections (42).

### Methionine salvage pathway

The methionine salvage pathway is another vital metabolic process of methionine in animals, plants, and bacteria. Within this pathway, SAM decarboxylase (SAMD) catalyzes the decarboxylation of SAM to generate decarboxylated SAM (dcSAM), which serves as a precursor for polyamines synthesis including spermine and spermidine. Methylthioadenosine (MTA), a by-product of this polyamine synthesis, undergoes phosphorylation by MTA phosphorylase (MTAP) resulting in the formation of 5’-methylthioribose-1-phosphate (MTR1P), eventually being converted back into methionine through a series of enzymatic reactions (**Figure 2**).

The key enzymes of the methionine salvage pathway are highly conserved across various immune cell types. Specifically, MTAP functions as the rate-limiting enzyme of methionine salvage pathway in this pathway and determines the level of its substrate MTA. This pivotal molecule occupies substrate site of SAM-dependent methyltransferase with SAM, suggesting that intracellular fluctuations in MTA levels may participate in the regulating SAM metabolism and methylation levels (43, 44). In addition, as a by-product of polyamine synthesis, MTA exerts a feedback inhibitory effect on ornithine decarboxylase, spermidine synthase, and spermine synthase (45). Based on these mechanisms, MTA plays relevant regulatory functions in immune cells, including lymphocyte activation, antibody response, NK cell cytotoxicity, and cytokine production (46–49). Serum MTA is implicated in hepatocellular carcinoma tumorigenesis by reducing the global chromatin accessibilities of CD8^+^ T cells and impairing the anti-tumor immunity (50).

### Catalytic cycle of methionine residues

Methionine possesses an electron-rich sulfur atom in its thioether side chain, which renders it prone to combine with the active substance and oxidized into two kinds of methionine sulfoxide (MetO) sulfoxide diastereomers: *S*-methionine sulfoxide and *R*-methionine sulfoxide. Intriguingly, this process is reversible as the oxidized methionine residues can be repaired back to methionine through the action of methionine sulfoxide reductase (MSR) A and MSRB (51). The catalytic cycle of methionine residues is regarded as the ultimate antioxidant defense mechanism in immune cells that crucial for the determination of cellular development and function. For example, genetic deletion of MsrB1 in LPS-stimulated macrophages suppresses the expression of anti-inflammatory cytokines and relieve inflammation (52). In addition to maintain the cellular redox balance, the catalytic cycle of methionine residues modulates the signal transduction pathways by the protein post-translational modifications, analogous to phosphorylation and dephosphorylation cycles (53, 54). The macrophages with *MsrB1*-depletion display the sustained oxidation of an exposed methionine residue M44 on glyceraldehyde 3-phosphate dehydrogenase (GAPDH), resulting in GAPDH aggregation, inflammasome activation, and subsequent increased IL-1β secretion (55).

### Regulation of methionine metabolism in innate immunity

#### Macrophage

Macrophages are highly sensitive to the exogenous stimuli including bacteria, viruses, protozoa, and other environmental challenges, which are following polarized into M1 or M2 subset. The metabolomics and transcriptomics data suggest that macrophages undergo a significant metabolic and transcriptional reprogramming upon activation, which drives the polarized phenotype and fulfills a broad range of functions (56, 57). In this context, several metabolic pathways (e.g., the serine synthesis pathway, one-carbon metabolism, and pentose phosphate pathway) in macrophages are activated by LPS stimulation. The altered pathways facilitate the production of SAM and maintain a high SAM/SAH ratio, which enhances H3K36me3 chromatin occupancy in *Il1b* gene and promotes the production of IL-1β (34). These findings confirm that cellular SAM levels regulate IL-1β secretion through methylation modification. Targeting SAM biosynthesis blockage may represent a potential therapeutic strategy for attenuating inflammation. However, previous studies have reported that methionine or SAM supplementation exerts an anti-inflammatory effect through suppressing the production of IL-6, tumor necrosis factor (TNF)-α, and IFN-β in LPS-stimulated inflammatory macrophages and microglial cells (58–60). Intriguingly, the inhibitory effects are mitigated by the treatment with UNC1999 (an inhibitor of H3K27me3) and 5-aza-2’-deoxycytidine (a DNA methyltransferase inhibitor) (unpublished data) (58, 60), suggesting methionine or SAM reduces the production of inflammatory factor in association with the histone and DNA methylation modification of specific genes. Furthermore, SAM serves as a CBS-specific agonist upregulates H_2_S content, which inhibits the overexpression of P2X7 receptor and activation of NOD-like receptor family pyrin domain-containing 3 (NLRP3) inflammasome in microglial cells after intracerebral hemorrhage, leading to the reduction of IL-1β (59). These differential effects of methionine metabolism on LPS-induced inflammation may be attributed to variations in the supplemental dosage of methionine or SAM, as well as the timing of LPS treatment.

Recent studies further suggest that these opposing effects of SAM on macrophage inflammation are mediated through multiple context-dependent mechanisms (61, 62). SAM acts as a universal methyl donor influencing both activating and repressive histone methylation marks (63). Increased SAM levels can enhance H3K4me3 at pro-inflammatory loci such as IL-1β, while simultaneously promoting H3K9me3 or H3K27me3 at loci like IL-6 and TNF-α, thereby suppressing their transcription (64). The final inflammatory outcome depends on the balance between these methylation events and the activity of specific histone methyltransferases and demethylases. In addition, SAM metabolism is tightly linked to redox homeostasis through the transsulfuration pathway (65). Under oxidative stress, SAM-derived homocysteine can be diverted toward glutathione (GSH) synthesis, limiting ROS accumulation and attenuating NF-κB–driven cytokine release (66). Conversely, excessive SAM accumulation without sufficient GSH conversion may promote ROS generation and activate the NLRP3 inflammasome, leading to IL-1β secretion (67). Temporal and dosage effects also modulate SAM’s dual roles that early or high-dose exposure tends to transiently activate inflammatory signaling, whereas prolonged or moderate supplementation restores methylation homeostasis and dampens inflammation (68). Collectively, these findings indicate that the dual pro- and anti-inflammatory actions of SAM arise from the integration of epigenetic, metabolic, and temporal regulatory layers that dynamically shape macrophage polarization. Nevertheless, methionine plays a pivotal role in driving the epigenetic modifications of key genes in M1 macrophage during the LPS-induced inflammation.

The metabolic reprogramming of methionine is also a crucial factor in the alternative activation and function of M2 macrophages. Mechanistically, the SAM derived from one carbon metabolism increases the promoter abundance of H3K27me3 of insulin-like growth factor 1 (IGF1), which in turn results in the inactivation of p38-dependent JAK-STAT1 axis and promotes the STAT6-mediated M2 polarization (69). In line with this, macrophages also utilize the apoptotic cell-derived methionine to sustain M2 macrophage function during efferocytosis. Methionine from the apoptotic cells is converted into SAM and decreased the expression of *Dusp4* through DNA methyltransferase-3A (DNMT3A)-mediated DNA methylation. This process induces the activation of p-ERK-Ptgs2-PGE_2_-TGF-β1 pathway and drives the macrophage-mediated tissue resolution (70). On the other hand, the methionine metabolism also supports tumor-associated macrophages (TAMs), the primary immune cells within tumor microenvironment, to polarize into M2 subtype for shielding the tumor immune surveillance. The MAT2A is highly expressed in TAMs that causes elevated activity of methionine cycle, which induces the expression of RIP1 through modulating the histone methylation modification (H3K4me3), and thus activating and maintaining the M2 phenotype of TAMs (71, 72). This observation implies that anti-MAT2A might block methionine catabolism by TAMs and enhance anti-tumor immunity.

**Figure 3:**
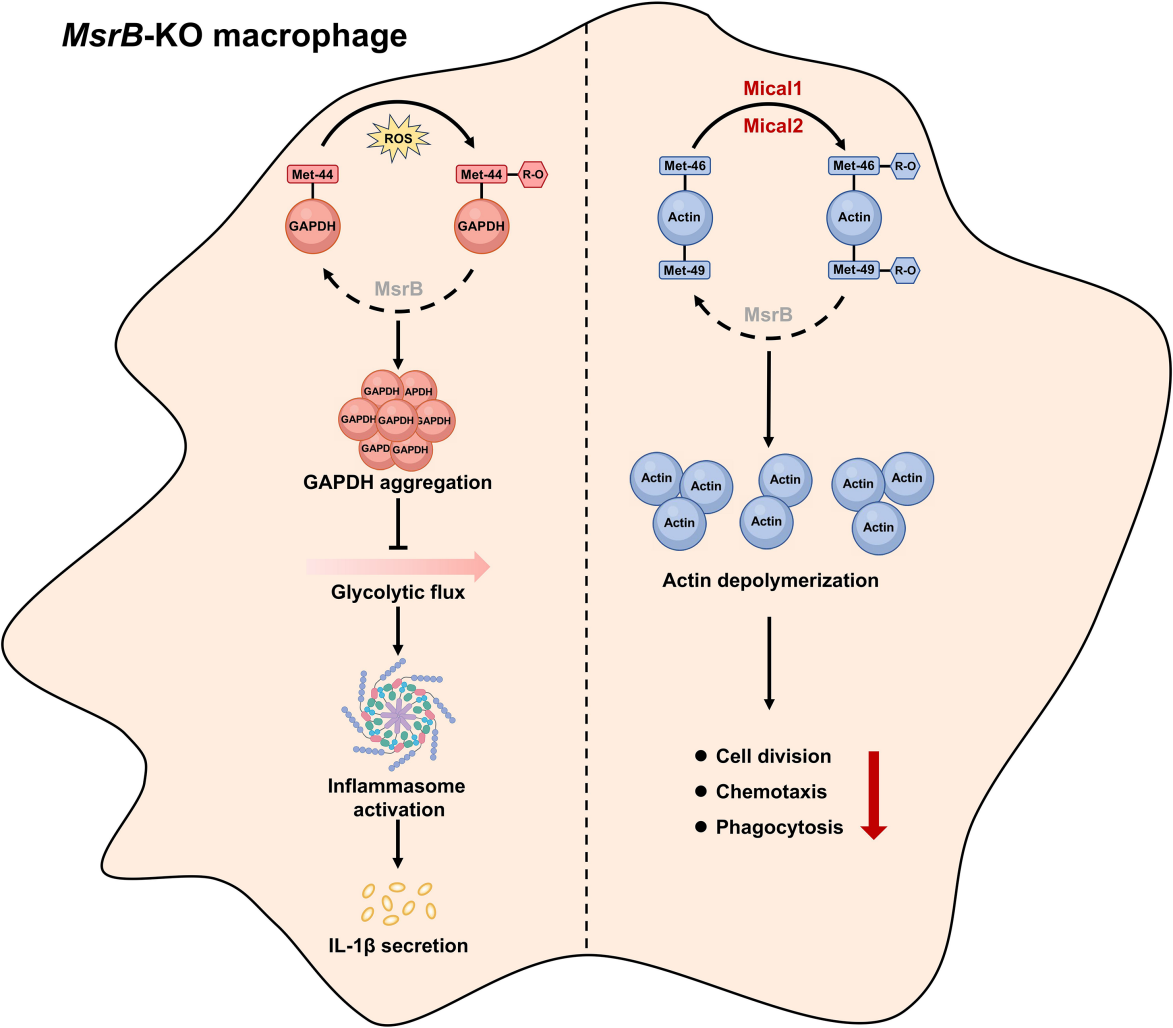
Methionine regulates macrophage function through the reversible oxidation and reduction of methionine residue. The function of macrophages is regulated by the reversible oxidation and reduction of methionine residues on specific proteins. The *MsrB* depletion inhibits the reduction of *R*-methionine sulfoxide exposed on GAPDH and actin, which results in the GAPDH aggregation or actin polymerization thereby regulates the inflammasome activation and immune response of macrophages.

As previously mentioned, the oxidation and reduction of methionine residue is a kind of reversible posttranslational modifications that modulates the protein functions and diverse biological processes. In macrophages, an exposed methionine residue (Met44) on glyceraldehyde 3-phosphate dehydrogenase (GAPDH) is identified to have critical roles in controlling the inflammatory activation (**Figure 3**). Specifically, the continuous oxidation of Met44 on GAPDH caused by *MsrB1* depletion induces the aggregation of GAPDH, activation of inflammasomes, and subsequent increase in secretion of IL-1β. However, this process can be effectively prevented in the presence of normal levels of MsrB1 through the reduction of methionine sulfoxide on GAPDH (55). Unfortunately, the biochemical and structural mechanisms underlying MsrB1’s specific recognition and reduction of *R*-methionine sulfoxide on Met44 remain elusive. Additionally, another two methionine residues (Met46 and Met49) on actin can also be specifically oxidated into *R*-methionine sulfoxide by monooxygenase Mical1 and Mical2 and reduced back to methionine by MsrB1, which supports the disassembly and assembly of actin to regulate cell division, motility, and signaling in macrophages (73) (**Figure 3**). Considering the impact of methionine residue redox regulation on key protein activation and macrophage function, manipulating its redox status holds promise as a viable strategy for addressing inflammatory diseases like sepsis.

### Dendritic cells

DCs are a class of professional antigen presenting cells that play central roles in both adaptive and innate immunity. DCs express multiple pattern recognition receptors, which are activated by pathogen-associated molecular patterns or damage-associated molecular patterns to induce immune tolerance or response (74–76). Similarly to its effect on macrophages, the oxidation and reduction of methionine residue act as crucial regulators for DC-related immune response. DCs from *MsrB1*-deficient mice, upon IL-4 stimulation, exhibit diminished pSTAT6 levels, leading to reduced IL-12 production and impaired antigen presentation of DCs (77). IL-4 binds to the type I receptor IL-4R, activating downstream protein kinase JAK1 and JAK3, which subsequently phosphorylate the transcription factor STAT6 (78). However, the specific targeting proteins and methionine residue sites involved in this context have yet to be identified.

There is currently a lack of specific publications regarding the direct influence of methionine in DCs. Nevertheless, several studies have elucidated that cysteine plays a crucial role in DC-mediated T cell response. As naïve T cells lack both cystathionase (which converts homocysteine to cysteine) and X_c_^-^ transporter (which imports cystine for cysteine production), they acquire their metabolic cysteine source depend on DCs or other antigen presenting cells (79). Consequently, several tumor-associated cells can suppress the antitumor immunity through inhibiting the cysteine biosynthesis. For example, myeloid-derived suppressor cells compete the extracellular cystine with DCs and mesenchymal stem cells restrain the expression of cystathionase of DCs, both of which suppress the functions of T cells within tumor microenvironment (80, 81). It would be intriguing to investigate whether supplementation of cysteine could rescue immune suppressions induced by tumor-associated cells and whether adequate cysteine levels in DCs enhance the ability of T cells to eradicate tumors.

### Natural killer cells

NK cells are innate-like lymphocytes with cytotoxic properties that recognize and eliminate invading tumor cells and virus infected cells, while simultaneously produce diversified pro-inflammatory cytokines or chemokines to recruit and activate other immune cells (82, 83). Early studies demonstrated that a methionine restriction diet inhibits the NK cell cytotoxicity and immune activity *in vitro*, but does not affect the percentage of splenic NK cells (84). Despite the results described above, there is currently a lack of research investigating how methionine metabolism mediates NK cell function. However, as early as 1984, it was revealed that methionine metabolite MTA suppresses the activity and cytotoxicity of NK cells through inhibiting transmethylation reaction rather than stimulating cyclic adenosine monophosphate (cAMP) production (85). Indeed, the MAT2A expression has been proven to be positively associated with the activation and cytokine production of NK cells, and the histone methyltransferase inhibitors significantly reduce the CD107a expression on the surface of NK cells (49, 86). In addition, MTA interferes downstream CD16 signal cascade for activation of NK cells, including PI3K-AKT-S6, MAPK-ERK, and NF-κB pathways (49). These data suggest that methionine actions in NK cell function involve both histone methylation modification and NK cell signaling activation.

### Regulation of methionine metabolism in adaptive immunity

#### T lymphocytes

Generally, one major alteration taken place during activation and differentiation of T cells is metabolic reprogramming, which provides sufficient metabolic substrates for this crucial biological process (87, 88). It has been demonstrated that activated T cells upregulate the expression of methionine transporters SLC7A5 and sustain a sharply elevation in methionine uptake from the external environment following antigen stimulation (9). Methionine restriction has limited T cell activation and inhibited the expansion of Th17 cells *in vivo*, while inhibition of MAT2A or deletion of intracellular SAM pools induces apoptosis in leukemic T cells (12, 89, 90). On the contrary, adequate methionine is essential for the survival of T-regulatory (Treg) cells in the absence of IL-2 (91). Collectively, these compelling observations suggest that cellular methionine attainment modulates the status of T cells.

**Figure 4:**
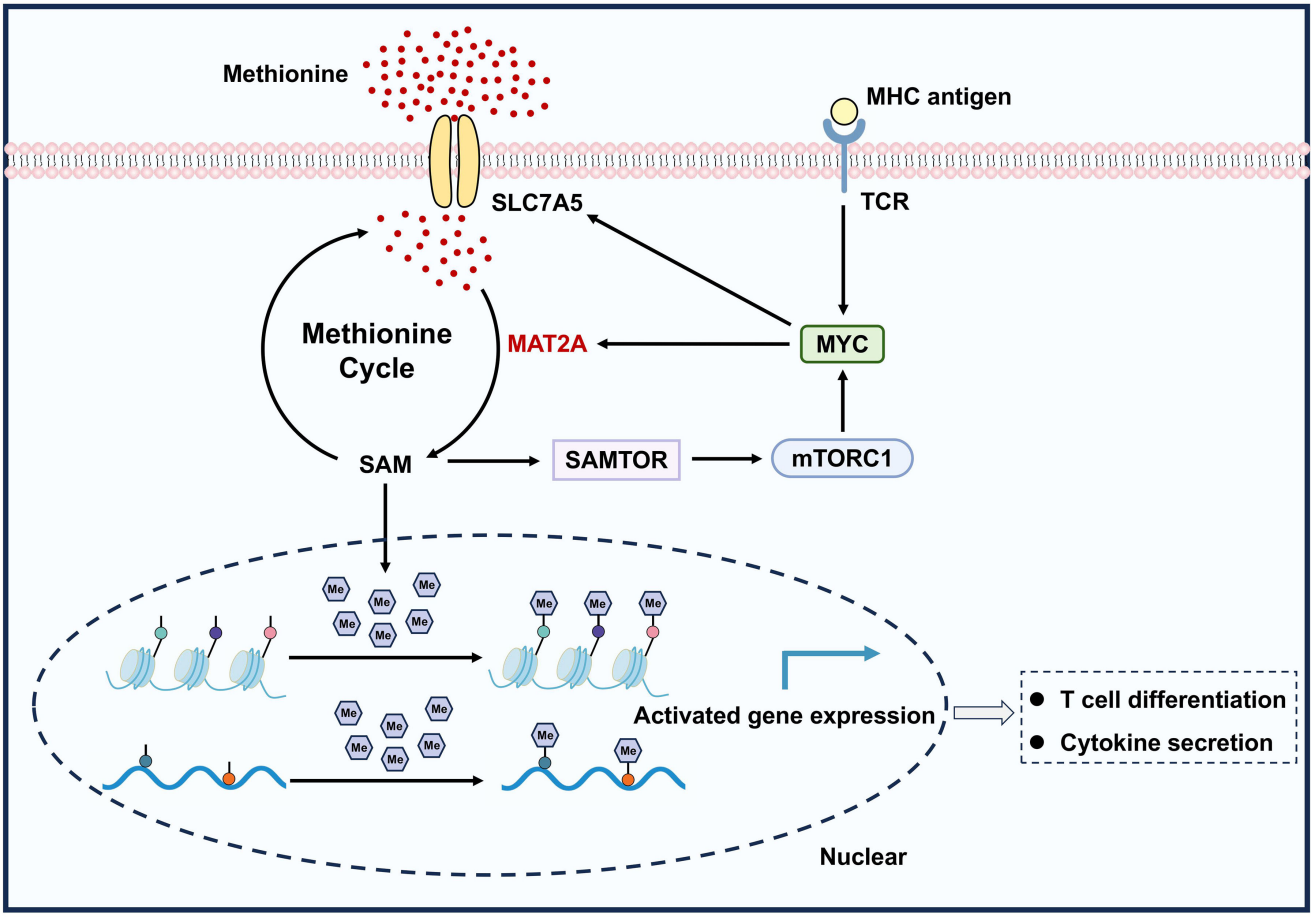
Methionine metabolism supports the activation and function of T cells. The protooncogene MYC is upregulated upon activation of T cell receptor (TCR) signaling by major histocompatibility complex (MHC) antigens, which enhances methionine uptake through upregulating the expression of SLC7A5. Methionine is then converted into SAM in the catalysis of MAT2A and provides methyl groups for RNA and histone methylation. Meanwhile, SAM in turn activates MYC via the SAM-SAMTOR-mTORC1-MYC axis, which further stimulates the SAM production by controlling the MAT2A expression. The SAM-dependent methylation participates in the epigenetic modification of key genes that contributes to the differentiation and function of T cells.

The phenotypic changes of T cells are important to perform their specific functions in immune response, which accompanied by distinct gene-expression programs. Methylation is one of the most important epigenetic modification types that regulates the gene expression through modulating the chromatin accessibility without modifying underlying genetic code (92, 93). Interestingly, it has been unconcealed that epigenome is dynamically regulated with the changes in nutrients, known as nutritional epigenomics (94, 95). As the immediate precursor of methyl donor SAM, methionine shapes the development and differentiation of T cells through driving the methylation modification. For example, the elevated methionine import promotes both RNA methylation (N6 adenosine and 5-methylcytosine) and histone methylation (H3K4me3 and H3K27me3) of CD4^+^ T cells, enabling epigenetic reprogramming and regulating proliferation and differentiation of T cells (9) (**Figure 4**). However, acute methionine deprivation inhibits histone methylation in activated CD8^+^ T effector cells (H3K4me3) and CD4^+^ Th17 cells (H3K4me3, H3K27me3, and H3K36me3), and these modifications influences the expression of key genes includes *Il17a*, *Batf*, and *Cd5l* (12). Apart from the acute impact on T cell function, methionine also has an enduring effect on T cell memory that relies on epigenetic modification. In CD8^+^ T cells, autophagy deficiency alters the glucose metabolism and causes cellular SAM reduction, which transcriptionally reprograms the CD8^+^ T cells towards effector memory (loss of H3K27me3 and gain in H3K4me3) (96). It is precisely because of the important role of methionine metabolism in methylation process, the tumor cells could hinder the methionine uptake of CD8^+^ T cells through highly overexpressing methionine transporter SLC43A2, resulting in the loss of H3K79me2 and defective STAT5 signaling and hence impairs T cell immunity (10). Significantly, although methionine is identified as an important factor of nutritional epigenomics for maintaining T cell function, the tumor-derived or exogenous SAM metabolite also reprograms chromatin accessibilities of CD8^+^ T cells, leading to T cell dysfunction (50). The exhaustion markers in CD8^+^ T cells treated with SAM are similar among those with mock treatment at day 3, the presence of T cell dysfunction only increased at day 7 and 14 (50). Collectively, methionine has great importance in the activation and effector function of T cells, but persistent SAM stimulation contributes to T cell dysfunction.

In addition to its role on cellular epigenetic modifications, methionine metabolism is also responsible for the proliferation and differentiation of T cells via activating of mammalian target of rapamycin (mTOR) pathway. The mTOR signaling has a central role in T cell fate decisions, and its regulatory functions in the differentiation of regulatory, effector, helper, and memory T cells are well documented in elsewhere (97–100). The mTOR signaling can be activated by three board categories of instructive signals, encompassing environmental cues (e.g., growth factors and immunoregulatory factors), immune signals (e.g., co-stimulation, antigens, and cytokines), and nutrients (e.g., essential amino acids and glucose) (98). Methionine mediates mTORC1 signaling in a Rag GTPase-dependent manner like leucine and arginine. The metabolite SAM can bind to the special SAM binding protein SAMTOR (or C7orf60) and disrupt the association of SAMTOR-GATOR1 complex, a negative regulator of mTORC1 pathway, thus activating the mTORC1 (101, 102). Simultaneously, the mTORC1 activation in turn increases the expression of MAT2A via the binding of downstream transcription factor MYC, which augments the m6A modification and protein synthesis rate for anabolic cell growth (103). Totally, these results do support the current belief that methionine metabolism positively mediate the proliferation and differentiation of T cells through mTORC1 pathway (**Figure 4**).

### B lymphocytes

B lymphocytes are an important part of adaptive immunity, which responsible for the antibody production and antigen presentation to T cells (104). The timely studies have uncovered methionine metabolism also has a profound effect on the differentiation of B cells and shares the similar mechanism with T cells. Intracellular methionine level of CD19^+^ B cells is largest increased after CpG/IFN-α/BCR crosslinking simulation, and methionine commits the differentiation of CD19^+^ B cells through driving the histone modification H3K27me3 of BACH2 (a vital transcriptional factor for B-cell differentiation), and this process refers to activation of spleen tyrosine kinase (Syk) or mTORC1 signaling and up-regulation of expression of methyltransferase enhancer zeste homolog 2 (EZH2) (105). However, there still has several questions are unclear in the current research. Although inhibition of either Syk or mTORC1 signaling both down-regulates the expression of EZH2, the interaction of Syk and mTORC1 signals in inducing EZH2 expression has not been fully elucidated. Syk is one of the key downstream components in the B cell receptor (BCR) signaling that response to B cell differentiation (106). As observed in malignant B cells, Syk depletion has significantly decreased the phosphorylation of p70S6K (an important effecter of mTORC1) (107), it indicates Syk is a critical upstream regulator in mTOR activation. Moreover, mTORC1 has been demonstrated to control the protein expression of EZH2, but not transcript expression, in both glioblastoma tumor cells and follicular lymphoma (108, 109). Therefore, the EZH2 expression is up-regulated via the activation of Syk and mTORC1 signals successively in CpG/IFN-α/BCR simulated B cells. Another compelling question is the specific role of methionine in driving histone modification of BACH2, whether via participating in the mediation of BCR-Syk-mTORC1-EZH2 or only providing methyl for the histone modification. An interesting observation is that methionine depletion has inhibited the EZH2 expression significantly in CpG/IFN-α/BCR simulated B cells (105), and the methionine level determines the mTORC1 activation as we described above, suggesting that mTORC1 is the mid-mediator of methionine induced EZH2 expression. However, apart from methionine, other amino acids, such as glutamine, arginine, and tryptophan, also response for the activation of mTORC1 (110), while they have little effects on the B cells differentiation when compare with methionine. Consequently, methionine functions as a specific amino acid in regulating B cells differentiation not only through activating mTORC1 signals but also exerting other physiological functions, such as provides methyl for epigenetic modification of key genes.

The relevance of methionine metabolism in B cells is also highlighted in several pathogen infection models. For instance, Epstein-Barr virus (EBV) would remodel the methionine metabolic pathway of infected B cells, which upregulated the methionine transporters on the plasma membrane of B cells (111). EBV then utilizes the methionine metabolic pathway of host to methylate EBV oncogenes, which alters the infected B cell biology and eventually transforms the B cells into lymphoblastoid B cells for the immunoevasion (112). This implicates methionine nutritional intervention as potential anti-EBV infection strategies.

**Figure 5:**
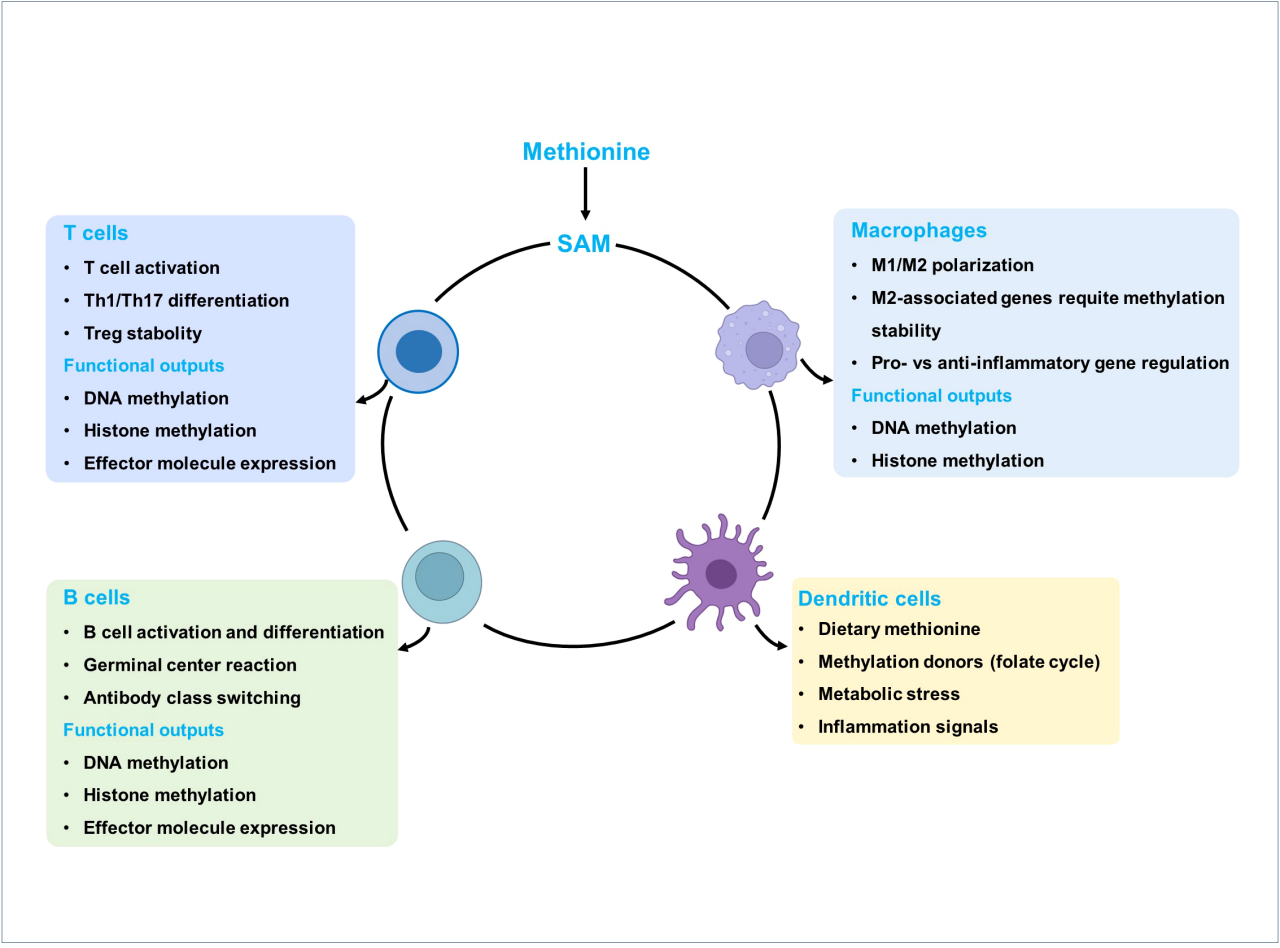
Overview of methionine metabolism and its regulatory roles in immune cell fate and function. Methionine enters the methionine cycle and is converted to S-adenosylmethionine (SAM), the universal methyl donor that supports DNA and histone methylation. Methionine metabolism affects the epigenetic regulation of various immune cell types through SAM, including T cells, B cells, macrophages, and dendritic cells. These epigenetic modifications modulate immune-cell activation, differentiation, effector functions, and inflammatory responses. External cues, such as dietary methionine supply, methyl donors, metabolic stress, and inflammatory signals, further shape methionine metabolism and thereby regulate immune responses.

Methionine enters the methionine cycle and is converted to SAM, the universal methyl donor that supports DNA and histone methylation. SAM is subsequently transformed into SAH and then to homocysteine, which can either be remethylated back to methionine or shunted into the transsulfuration pathway to generate cysteine and GSH. Methionine metabolism affects the epigenetic regulation of various immune cell types through SAM, including T cells, B cells, macrophages, dendritic cells, and natural killer cells. These epigenetic modifications modulate immune-cell activation, differentiation, effector functions, and inflammatory responses. External cues, such as dietary methionine supply, methyl donors, metabolic stress, and inflammatory signals, further shape methionine metabolism and thereby regulate immune responses (**Figure 5**).

## Role of methionine metabolism in cancer development

### Elevated methionine demand in cancer cells

Cancer cells have an absolute metabolic requirement for methionine, and the elevated exogenous methionine uptake occurs in the tumor-initiating cells because of its highly improved methionine cycle activity (113). Numerous studies documented that a variety of cancer cells overexpressed the methionine transporters (e.g., SLC43A2, SLC7A5, and SLC6A14) to uptake and outcompete methionine resource with immune cells (10, 114–116). This elevated methionine uptake of cancer cells can probably be attributed to the multifaceted factors. Firstly, methionine functions as the raw material for protein biosynthesis and supports the proliferation of cancer cells. In addition, methionine metabolism acts as a crucial branch polyamine biosynthesis that imperative for the rapid proliferation of cancer cells, and SAMD functions as the rate-limiting enzyme in this metabolic pathway directly regulated by the oncogene MYC (117–119). Moreover, numerous studies reveal that the *CDKN2A* locus (encodes the tumor suppressor p16) on chromosome 9p21 is frequently lost in 15% of all cancer cell subsets, and interestingly, the adjacent *MTAP* locus is co-deleted in 80-90% of these cancer cells (120, 121). The *CDKN2A* loss leads to the transformation of normal cells into cancerous tumor cells, while *MATP* loss causes the methionine salvation deficiency in cancerous cells. Thus, the impaired methionine biosynthesis increases the dependence of cancer cells on exogenous methionine.

Much attention has been placed on the epigenetic modification of methionine on cancer-associated cellular processes, and two major aspects for methionine-mediated methylation on cancer development have been reported. On the one hand, the cancer cells escape the anti-tumor immunity through the methylation of immune checkpoint molecules. In the colorectal cancer cells, methionine-derived SAM has promoted the RNA m^6^A methylation level and the translation of PD-L1 and V-domain Ig suppressor of T cell activation (VISTA) (122). Meanwhile, insufficient extracellular methionine supply in the tumor microenvironment augments PD-1 expression through decreasing the H3K79me2 level in CD4^+^ T cells (123). On the other hand, methionine flux also supports the cancer cells to escape from cyclic GMP-AMP synthase (cGAS) mediated anti-tumor immunity in the methylation dependent manner. The methylated cGAS can binds with UHRF1 to promote cGAS chromatin sequestration and loss its activity, and eventually suppress the IFN-I responses (124). Moreover, high methionine flux also supports the cancer cells to evading pyroptosis, which maintains the genome-wide hypermethylation that regulates the genes refer to metal ion transporter, SMADs, and cGAS-IFN-1 signaling cascade (125).

### Targeting methionine metabolism for cancer therapy

**Figure 6:**
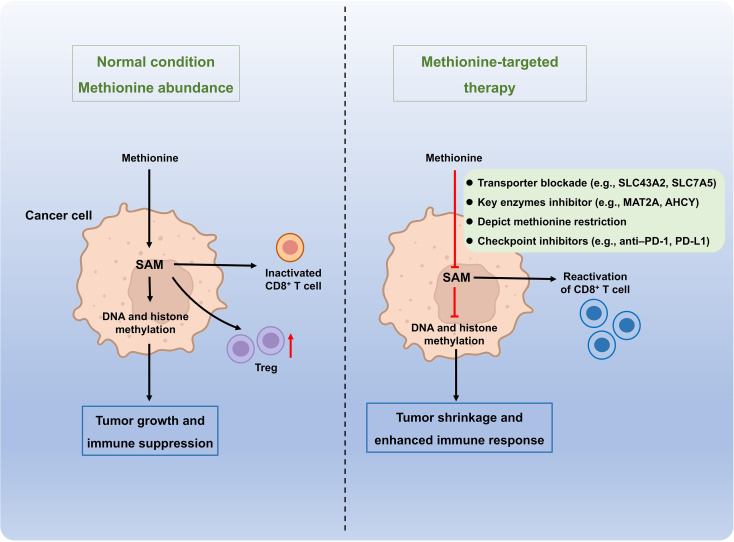
Schematic overview of the role of methionine metabolism in cancer progression and potential therapeutic targets. Methionine metabolism plays a central role in supporting tumor growth and immune evasion by fueling one-carbon metabolism, polyamine synthesis, and methylation reactions. Cancer cells exhibit elevated methionine uptake through transporters such as SLC43A2 and SLC7A5, which provide substrates for the methionine cycle and downstream methyl donor S-adenosylmethionine (SAM). Increased SAM availability promotes DNA and histone methylation, leading to epigenetic silencing of tumor suppressor genes and modulation of immune signaling. In parallel, methionine-derived intermediates support redox balance and nucleotide biosynthesis essential for rapid proliferation. Targeting methionine metabolism can therefore suppress tumor growth and restore antitumor immunity. Potential therapeutic strategies include: inhibition of methionine transporters (e.g., SLC43A2, SLC7A5); blockade of key metabolic enzymes (e.g., MAT2A, AHCY, and MTR); dietary methionine restriction (MR) to limit systemic methionine availability; combination therapies integrating MR or metabolic inhibitors with immune checkpoint blockade (e.g., anti–PD-1/PD-L1). Collectively, these interventions aim to reprogram tumor metabolism, enhance immune activation, and improve the efficacy of cancer therapy.

Due to its crucial role for the cancer progression, methionine metabolism has been a potential therapeutic target in cancers. As illustrated in **Figure 5**, methionine metabolism promotes tumor progression through enhanced SAM-dependent methylation and immunosuppressive reprogramming. Emerging therapeutic strategies such as inhibition of methionine transporters (e.g., SLC43A2, SLC7A5), blockade of key metabolic enzymes (e.g., MAT2A, AHCY, and MTR), depict methionine restriction, and checkpoint inhibitors (e.g., anti–PD-1, PD-L1), offer promising avenues to restore antitumor immunity and improve cancer treatment outcomes (**Figure 6**). The *in vitro* experiments evidenced methionine starvation, even if the short-term starvation, contributes to the severe disruption of cancer development, including tumor-initiating ability, tumor growth, and cancer cell migration (113, 126). In addition, numerous pre-clinical trials have demonstrated that dietary methionine restriction, a diet characterized by complete or partial removal of methionine, ceases tumor growth and enhances the efficacy of cancer therapies in many cancer types, such as breast cancer, prostate cancer, glioma, sarcoma, colorectal cancer, and melanoma (127, 128). Interestingly, humans who received the methionine restriction diet show a similar metabolic profile with the methionine-deprived tumor-bearing mice (129), and one prediction from this finding is that methionine restriction in cancer patients is also likely to exert anti-tumorigenic effects as in murine models. Remarkably, dietary methionine restriction has a more significant influence on the prevention of colorectal cancer than treatment (129), and thus the dietary methionine restriction intervention is currently act as an auxiliary means of chemotherapy and radiation (**Table 1**).

**Table 1:** The summary of methionine-rich and methionine-deprived foods.

Category	Methionine-rich foods	Methionine-poor foods	References
Animal-based	Eggs, fish, poultry, beef, dairy products	/	(130–132)
Plant-based	Brazil nuts, sesame seeds, soybeans, lentils, oats	Fruits (apple, orange, banana), most vegetables (spinach, lettuce, tomato), rice, corn, potatoes, berries, leafy greens, potatoes	(133–136)

Beyond restricting the dietary methionine uptake, methionine metabolic enzymes are another attractive target for cancer therapy. A number of studies have found that expression of MATs is higher in various cancer tissues than that in normal tissues, and the inhibitors of methionine adenosyltransferase MAT2A receive extensive attention in the cancer therapy (31). Inhibition of MAT2A by shRNA or inhibitors has reduces the proliferation of several cancer cell lines through inducing the global depletion of H3K36me3, and MAT2A knockdown prolonged the median survival in DIPG13p model mice (137). Unfortunately, although MAT2A is indeed an excellent potential target for cancer therapy and multiple MAT2A inhibitors are identified in recent years, there still lacks the mature MAT2A inhibitor drug for the clinical application of the broad-spectrum cancers due to various shortages. For example, the FIDAS agents are a class of substrate competitive inhibitor of MAT2A that possess activity against various liver and colorectal cancer cell lines, but its carbon-carbon double bond could be oxidated and cause the off-target biological effects (138). Surprisingly, MAT2A inhibition exhibits a gratifying result in the *MTAP*-deleted cancers. As mentioned before, MATP loss of cancer cells leads to the accumulation of its substrate MTA and further inhibits the activity of arginine methyltransferase, which causes the heightened susceptibility to the PRMT5 depletion and impairs the cancer cell viability through synthetic lethality machinery (139, 140). This MTAP deletion-induced vulnerability is extend to the MAT2A, the upstream enzymes of PRMT5, and three kinds of MAT2A inhibitor (AG-270, S-095033, and IDE-397) are currently in the phase I clinical trials for the therapy of MTAP-deleted cancers (141). In future studies it will be necessary to continue to reveal the biological characteristics of MAT2A function for further exploring the potential of MAT2A-targeted cancer therapies.

## Future directions

Despite significant progress in elucidating the role of methionine metabolism in immune regulation and tumor biology, many questions remain unanswered. Future studies should focus on integrating methionine-targeted interventions with established immunotherapies, such as immune checkpoint blockade and adoptive T cell transfer, to enhance antitumor efficacy. Moreover, the development of selective inhibitors that modulate methionine transporters or key enzymes in the methionine cycle could offer novel therapeutic opportunities while minimizing systemic toxicity. Dietary methionine restriction, when combined with pharmacological strategies, represents another promising yet underexplored approach to reprogram the tumor microenvironment and improve treatment outcomes. Comprehensive clinical trials are needed to define optimal dosing regimens, identify patient populations most likely to benefit, and elucidate long-term metabolic and immune consequences of methionine modulation.

## Concluding remarks

The biosynthesis and catabolism of amino acids constitute an essential component of cellular intermediary metabolism, and compelling results have emphasized the crucial role of methionine metabolism in regulating metabolic and immune response. As such, reprogramming methionine metabolism may offer promising strategies for preventing or ameliorating the autoimmune diseases and associated pathologies. For example, reducing the availability of methionine resulted in a slower onset and progression of experimental autoimmune encephalomyelitis disease (12). In addition to directly acting on methionine availability, the transporters, metabolic enzymes, and sensors of methionine represent potential targets for therapeutic intervention. However, it is undoubtedly that there exist significant knowledge gaps about methionine metabolism on diverse immune cell types and functions. To date, apart from the macrophages, T cells, and B cells, the immune regulatory role of methionine metabolism in other immune cell types is not fully elucidated. Moreover, while the extracellular transport of methionine has been elucidated in immune cells, the mechanisms by which methionine traffics from the cytosol to key organelles such as mitochondria and lysosomes, as well as its regulatory role in organelle function, remain enigmatic. Overall, targeting metabolic pathways of methionine in immune cells represents a valuable strategy for enhancing or inhibiting immune responses, and gaining deeper insights into the intricate mechanisms of methionine metabolism in immune cells is expected to yield significant therapeutic advantages.
